# Perspectives and Influences of Intergenerational Caregivers on Cariogenic Feeding Practices in Childhood: A Qualitative Study

**DOI:** 10.1111/ipd.13320

**Published:** 2025-05-07

**Authors:** Su Lin Quak, Huei Jinn Tong, Catherine Hsu Ling Hong, Mary Foong Fong Chong, Monty Duggal, Zubair Amin, Xiaoli Gao

**Affiliations:** ^1^ Faculty of Dentistry National University of Singapore Singapore Singapore; ^2^ Health Promotion Board Singapore Singapore; ^3^ Saw Swee Hock School of Public Health National University of Singapore Singapore Singapore; ^4^ Singapore Institute for Clinical Sciences (SICS) Agency for Science, Technology and Research (A*STAR) Singapore Singapore; ^5^ College of Dental Medicine Qatar University Doha Qatar; ^6^ Department of Paediatrics, Yong Loo Lin School of Medicine National University of Singapore Singapore Singapore; ^7^ Department of Neonatology, Khoo Teck Puat – National University Children's Medical Institute National University Hospital, National University Health System Singapore Singapore

**Keywords:** Chinese, feeding practices, grandparents, parents, preventive dentistry

## Abstract

**Background:**

Grandparental influences on child feeding practices that impact oral health remain under‐investigated.

**Aim:**

Explore perspectives and influences of intergenerational caregivers on cariogenic feeding practices in children.

**Design:**

This qualitative study is based on phenomenological approach. Data collection involved in‐depth interviews with parent(s) and grandparent(s) from the same family.

**Results:**

Thematic saturation was achieved after 26 interviews (13 families) with 13 mothers, 13 grandmothers and one grandfather. Three themes were generated: (1) *Intergenerational differences in milk feeding practices*—Grandparents had strong preference for formula milk. They advocated formula milk feeding beyond infancy and sometimes encouraged cariogenic comfort feeding habits; (2) *Transgenerational influences on snacking* practices—Parents' and grandparents' own cariogenic snacking habits encouraged the child's cariogenic snack intake. Grandparents occasionally used sugary treats to reinforce their special identity as grandparents; and (3) *Impact of new information sources* versus *grandparental input*—Information sources influencing child feeding practices differed between generations; parents relied on social media (rather than grandparental advice) while grandparents drew from past experiences. However, grandmothers who were actively involved in food preparation had considerable influence on child feeding practices.

**Conclusion:**

Practitioners and policymakers should actively engage grandparents to provide them with appropriate information on avoiding cariogenic feeding practices.

**Summary**

- Why this paper is important to pediatric dentists
  ○ Grandparents from contemporary intergenerational families who are actively involved in caregiving have a significant role in shaping child feeding practices.
  ○ Differences in child feeding beliefs and practices between intergenerational caregivers present challenges to the establishment of healthy feeding practices and the elimination of cariogenic feeding habits.
  ○ This study highlights the significance of family‐centric interventions for fostering healthy child feeding practices in intergenerational families.

## 1 Introduction

Early childhood caries (ECC) is preventable but remains the most common chronic childhood disease worldwide, with an increasing prevalence in some developed countries [1]. In Singapore, 17.8% children were affected by ECC at 2 years of age and 42.9% at 3 years of age [2]. Early intervention with a focus on infant to preschool‐aged children is warranted to mitigate the potentially devastating sequelae of ECC such as pain, emergency visits, high treatment costs and diminished child's and family's oral health‐related quality of life [3].

While ECC is a multifactorial disease, substantial epidemiologic evidence has demonstrated the significant role of dietary habits in the development of ECC [4]. Child feeding practices such as bedtime bottle feeding with sweetened beverages [5], early introduction of sweetened food and drinks [6], and frequent consumption of sugar‐added snacks [7] are well‐documented risk factors. Cariogenic feeding practices are common in Singapore; more than a third of children were put to sleep with a sweetened beverage at 12 months of age, and the majority used the bottle before sleep after 12 months [8].

While parents' influence on child feeding is well documented [9], grandparents and other caregivers also play a crucial role. Informal grandparental childcare is common in many cultures, and grandmothers, in particular, have been found to significantly influence child feeding practices. This is especially true in more hierarchical Asian societies, where they often serve as advisors to younger mothers [10]. In Singapore, 35% of grandparents over the age of 55 regularly care for their grandchildren [11], and in 17% of families, they were the primary decision‐makers regarding food [12]. Additionally, approximately one in five Singaporean families employs domestic helpers [13]. Various factors, including perceived roles, caregiving time and cultural influences, shape grandparents' approach to child feeding [14, 15]. Despite this, the specific impact of grandparents on cariogenic feeding practices remains under‐investigated. Only two studies, conducted in the United States, have been published using the same sample group [15, 16]. They explored mothers' perspectives on grandparental practices of offering cariogenic food and beverages to their grandchildren. However, they elicited only the mothers' narrative without input from the grandparents and did not explore cariogenic milk feeding practices.

The aim of this study was to explore the perspectives and influences of intergenerational caregivers on cariogenic feeding practics in early childhood. By understanding how cariogenic feeding practices are shaped within an intergenerational childcare setting, this study would address a public health issue affecting many young children and their families globally.

## 2 Materials and Methods

This was a qualitative research study using a phenomenological approach which explores a phenomenon from the perspective of those who have experienced it [17]. The idea is to develop new meanings and appreciations of the individual's lived experience to inform a researcher's understanding of that phenomenon. Data collection was through in‐depth interviews with the parent(s) and grandparent(s) from the same family who were involved in child feeding. Ethical approval was obtained from the National University of Singapore's Institutional Review Board (NUS IRB) (reference no.: 2020/01478). We followed the Consolidated Criteria for Reporting of Qualitative Research (COREQ) Checklist in reporting this study (Appendix A) [18].

Participants were recruited using a mix of purposive and snowball sampling strategies. Purposive sampling was carried out initially to ensure recruitment of a variety of families from different socio‐economic strata. Parental education level (university degree holders vs. non‐university degree holders) and working status (full‐time vs. non full‐time) were used in the sampling matrix to guide the recruitment of participants (Table 1). Participating families were recruited from a well‐child medical clinic affiliated to a tertiary hospital. A flyer was disseminated by the referring clinician and interested participants were approached face‐to‐face or over the telephone by a study team member. Following that, participants were concurrently recruited via word‐of‐mouth from existing study participants as part of the snowball sampling strategy. Recruitment ended when data saturation was reached.

**TABLE 1:** Sampling matrix.

Education level	Working status	
	Both parents working full‐time recruited/(target)	One or no parent working full‐time recruited/(target)
Both parents have university degree	4 families/(3–4 families)	4 families/(3–4 families)
One or no parent has university degree	4 families/(3–4 families)	1 family/(3–4 families)
Total	13 families/(12–16 families)	

The inclusion criteria were as follows:

- families with a first child aged between 12 and 36 months (in order to capture parents' first child feeding experience alongside grandparental involvement);
- at least one parent and grandparent were involved in caregiving for ≥ 10 h/week (similar to criteria used in a previous study) [19];
- at least one parent and grandparent in the same family consented to interview and audio recording; and
- Chinese race (to reduce variation due to cultural differences as a first exploratory study into this topic).

The family was excluded if the child had any medical, cognitive and/or developmental disorders that significantly affected feeding (e.g., receiving nasogastric or orogastric tube feeding or diagnosed with autism spectrum disorder).

We developed an interview guide (Appendix B) which was shaped by our research question and then refined through a thorough review of current literature on child feeding practices. Additionally, we drew upon the expertise of a multidisciplinary research team, which included professionals in dental public health, paediatric dentistry, health education, paediatrics and nutrition. The participants were asked about child feeding practices from birth to current times, with deeper exploration into perspectives and influences of intergenerational caregivers on cariogenic feeding practices. Pilot interviews were conducted with two parents and one grandparent and data from these pilot interviews were excluded from the study.

Socio‐demographic data and basic information on childcare and child feeding were collected via the short questionnaire (Appendix C) prior to the interview. Interviews were conducted separately to allow participants to share their views freely without any interference. Interviews were done face‐to‐face at the participant's home or over videoconferencing as per participant's preference, in either English or Mandarin. A single female interviewer (Author 1) who was formally trained in qualitative research methods and a native speaker of both English and Mandarin conducted all interviews. There was no pre‐existing relationship between the participants and the interviewer. The interviewer introduced herself as a children's dentist interested in studying the effect of feeding practices on dental caries. The goals of research and data confidentiality were explained and participants provided written informed consent. Each participant received S$30 (≈USD22) and a child toothbrushing set as tokens of appreciation for participation. Field notes were taken to capture the interviewer's raw thoughts and reflections. The interviews were audio recorded, transcribed verbatim and then coded in qualitative analysis software (NVivo version 12, 2022). Interviews conducted in Mandarin were translated to English. A randomly selected sample of translated interviews were cross‐checked by another native Mandarin speaking author (Last author) to ensure accuracy of translations.

Inductive thematic analysis, as described by Braun & Clarke, was used [20]. Under the inductive approach, themes emerged from data, without preconceived categories or theories. Multiple rounds of line‐by‐line coding were carried out by a single coder (Author 1). The key elements that were relevant to the area of inquiry were identified and labelled concretely by using either the informant's words (in vivo codes) or the words and concepts of the researchers' disciplines (in vitro codes). This process of open coding led to a clustering of substantive codes with similar contents into themes. Initial candidate themes were repeatedly revised and refined after discussions with the study team before the development of the final themes which best captured and conceptualised the data. Members aimed to avoid bias from pre‐structured understanding and maintained a self‐reflective attitude on data interpretation. They also considered competing explanations and alternative interpretations throughout the analysis.

## 3 Results

Of the 25 families screened, 13 families were recruited. The 12 families who were not recruited either did not fulfil the inclusion criteria (*n* = 6) or declined participation (*n* = 6). A total of 26 interviews were conducted with 13 families (13 mothers, 13 grandmothers and 1 grandfather). In one interview, a grandfather joined the grandmother at her request. Thematic saturation was reached after the 13th family and recruitment was stopped.

Recruitment targets in the sampling matrix were met except for the last category (i.e., non‐university degree parents who are not engaged in full‐time employment) (Table 1). Tables 2 and 3 depict the socio‐demographic characteristics and caregiving profiles, respectively. In most families, both parents were university graduates working full‐time, while the majority of grandparents were retired or not working. None of the grandparents had university degrees, which was uncommon for their age group in Singapore. Mothers, followed by grandmothers, were the primary caregivers and thus made up most of our participants. Fathers, grandfathers and domestic helpers played less central roles in caregiving. Most of the children (*n* = 8) attended formal childcare, a common practice in Singapore especially for children over 18 months old. Nearly half (*n* = 6) of the families lived in three‐generational households. Parents and grandparents who did not live together travelled regularly between the two homes with the child (possible due to the small size of the city‐state), making the differences of living together or separately less distinguishable.

**TABLE 2:** Socio‐demographic characteristics of the 13 recruited families.

	Parents (*n* = 13)		Grandparents (*n* = 14)^a^
Age (mean in years, range)	33.8 (23–43)		62.8 (49–73)
21–30	3		
31–40	9		
41–50	1		1
51–60			1
61–70			11
71–80			1
Gender			
Female	13		13
Male	0		1
Marital status			
Single	0		0
Married	13		12
Widowed	0		2
Highest education level	**Mother**	**Father**	
Primary	0	0	2
Secondary	2	1	9
Vocational education^b^	2	3	3
University degree	9	9	0
Working status	**Mother**	**Father**	
Full‐time	9	12	2
Part‐time	2	1	1
Not working	2	0	3
Retired	0	0	8
Child age (mean in months, range)	23 (13–36)		
12–17	4		
18–23	4		
24–36	5		

^a^ In one of the 13 families, two grandparents joined the interview. Hence, the total number of grandparents being interviewed was 14.

^b^ Vocational education includes Junior College, Polytechnic and Institute of Technical Education in Singapore.

**TABLE 3:** Caregiving profiles of the 13 recruited families.

Family number	Relationship of interviewees	Primary caregivers	Secondary caregivers	Child attends formal childcare	Live together in multigenerational home
01	Parent–Child	Mother, Grandmother	Father, Domestic helper	Yes	Yes
02	Parent–Child	Mother, Grandmother, Grandfather, Domestic helper	Father, Domestic helper	Yes	No
03	Parent–Child	Mother, Father	Grandmother, Grandfather, Domestic helper	Yes	Yes
04	Parent–Child	Mother, Father, Grandmother	Grandfather	No	No
05	Parent–Child	Mother, Grandmother	Father	Yes	Yes
06	In‐laws	Grandmother	Mother, Father, Grandfather	Yes	No
07	Parent–Child	Mother	Grandmother	Yes	Yes
08	In‐laws	Mother, Father, Grandmother, Grandfather	—	Yes	No
09	In‐laws	Mother	Father, Grandmother	Yes	No
10	Parent–Child	Grandmother	Mother, Father	No	No
11	Parent–Child	Mother, Grandmother, Domestic helper	Father	No	No
12	Parent–Child	Mother, Grandmother	Father, Grandfather, Aunts	No	Yes
13	In‐laws	Mother, Father	Grandmother, Grandfather	No	Yes
Overall	In‐laws (*n* = 4) Parent–Child (*n* = 9)	Mother (*n* = 11) Grandmother (*n* = 9) Father (*n* = 4) Grandfather (*n* = 2) Domestic helper (*n* = 1)	Father (*n* = 8) Grandmother (*n* = 4) Grandfather (*n* = 5) Domestic helper (*n* = 3) Mother (*n* = 2)	Yes (*n* = 8) No (*n* = 5)	Yes (*n* = 6) No (*n* = 7)

Each interview lasted an average of 61.2 min (range: 34–99 min). No repeat interview was carried out. All transcripts were deemed to have sufficient clarity for analysis and not returned to the participants for comments or corrections.

Three themes describing the perspectives and influences of intergenerational caregivers on child feeding practices were generated from the data. Participants were labelled: P—Parent, GP—Grandparent and Number—family number according to recruitment sequence.

### 3.1 Intergenerational Differences in Milk Feeding Practices

#### 3.1.1 Generational Perspectives on Choice of Milk in Infancy and Early Childhood

There was a distinctive divide between parents and grandparents regarding milk feeding beliefs and practices. Firstly, a generational change in infant milk feeding practices was evident, where there was a shift towards feeding breastmilk rather than formula milk in infancy. Grandmothers attributed this to improved societal support for breastfeeding and advancements in equipment for breastmilk expression (e.g., breast pumps) compared to the past.

Last time, if you wanted to breastfeed, you had no equipment; you had to manually express it (breastmilk) out with your hands. Now, you have machines to help you pump out, everything is just so much better. (GP12)

While grandparents showed general awareness about the nutritional and psychosocial benefits of breastfeeding, their support for breastfeeding appeared to be short‐lived. They commonly perceived breastfeeding (or breastmilk expression) to be unnecessarily stressful for mothers especially after they returned to work.

Of course breastmilk is better. But cannot drink it for too long, because the mother needs to work. It's not convenient. (GP02)

Some grandparents expressed doubts about the continued nutritional value of breastmilk after the introduction of solid foods. Others were not convinced that the benefits of breastmilk justified the effort. In certain families, grandparents entirely discouraged breastfeeding due to the belief that formula milk was more nutritious than breastmilk. Negative grandparental attitudes towards breastfeeding were a source of stress for mothers who wanted to continue breastfeeding.

My father‐in‐law was saying that I cannot breastfeed *lah [a filler word]*. She (the child) has no nutrients. She has to take powder (milk). (P03)

Beyond infancy, grandparents continued to advocate for formula milk feeding and often discouraged parents from giving the child cow's milk. Grandparents viewed formula milk more favourably, believing it contained vitamins and supplements that were essential for childhood years. They often did not consider the sugar content when choosing the type of milk to feed. Parents reported that this strong grandparental preference for formula milk stemmed from their past experiences with child feeding. On the contrary, parents showed more concern about the potential cariogenicity of formula milk. They were more knowledgeable of current recommendations regarding transition to cow's milk after age one and were more likely to implement these changes.

And one thing I don't like about formula (milk) is the added sugar that they have in it. (P04)

Yeah, to them (grandparents) is like, just give formula milk. Why need to give cow's milk? They think that like cow's milk is not as nutritious as formula milk. Because all along they gave us formula milk also, so they didn't know that actually after one year old, we can transition to cow's milk. So they were a bit skeptical. (P12)

#### 3.1.2 Beliefs Towards Comfort Feeding

Comfort feeding (i.e., sleeping with a milk bottle or latching on breast) was a common practice that extended into the toddler years for some families. Milk feeding was seen as an easy and convenient way to pacify a fussing child in times of need, especially at bedtime. Parents tended to be more aware than grandparents of the cariogenicity of these comfort feeding behaviours. Nevertheless, some prioritised the convenience of putting the child to sleep with milk over dental health. This was more evident in families where children experienced sleep difficulties, resulting in a higher level of caregiver stress and desperation.

I just think that it's tiring because they don't sleep through the night (…) We fight so many battles, right? This is not one of the top ten battles! (P01)

Grandparents frequently believed that formula milk was necessary to satiate the child before sleep. They were often unaware about the potential detrimental effects on the child's dentition and showed indifference towards comfort feeding habits, believing that bedtime bottle‐feeding was a natural feeding pattern for children.

Every baby is like that, they need milk before they sleep. If not, how do they fall asleep? You must let her drink until she's full then she can sleep properly. (GP10)

In some families with older children (> 18 months), intergenerational tension emerged due to differences in comfort feeding beliefs, particularly bedtime bottle‐feeding. Mothers felt that grandparents misinterpreted the child's cries as signs of hunger and were quick to pacify the child with milk. Parents expressed disapproval towards such grandparental feeding practices and were more reluctant to encourage continued reliance on milk for comfort due to perceived negative implications on dental health (dental caries) and sleeping habits (e.g., lack of ability to self‐soothe).

I had to stop them (grandparents) from feeding them milk for comfort. (…) They were not agreeable! They felt like, how can you listen to your children cry and not feed them, you know, (when) they're hungry! (P02)

In some families where grandparents were responsible for looking after the child at night, whether living together in three‐generational households or when the child stayed over at the grandparents' home, parents struggled to change grandparental perceptions about bedtime comfort feeding. Parents eventually accepted that they had limited control over feeding practices that occurred under the grandparents' care.

We didn't want to interfere. We knew that he should be cutting off his (bedtime) milk feeds. (…) But with the slightly senior folks, they are more creatures of habit right. (…) They don't understand. Some battles are meant not to be fought, so we just left it as that. (P08)

### 3.2 Transgenerational Influences on Child Snacking Practices

#### 3.2.1 Family Food Values and Influences

The parents' and grandparents' own snacking practices had considerable influences on the child's snacking habits. Children in families where caregivers did not buy or consume cariogenic snacks were unlikely to consume them. Conversely, caregivers who snacked on cariogenic food would typically offer them to the child. Despite knowing that such snacks were detrimental to the child's dental health, participants expressed, with some guilt, their role in influencing the child's cariogenic snack intake.

Because I snack a lot, and sometimes they see me snacking and they end up eating my snacks. Which is not exactly the kind of snack that I would buy for them. (P01)

Grandparents also had an indirect influence on child snacking habits via the transmission of food values and practices through the parents to the child. An example from GP06 illustrated how grandparental values of regulating sugary drink intake were ‘passed down’ to the grandchildren through first influencing parents' own practices:

Even for my own children, in the past, they could only start drinking these sweet drinks in secondary school. (…) So it cultivated into this habit, so they also don't offer these things to their own children. (GP06)

Food preferences and practices were generally more aligned among family members with direct parent‐and‐child relationships. However, notable differences emerged between spouses and in‐laws, who often held fundamentally different beliefs and practices. In such situations, some families struggled to reach a consensus on feeding rules and practices for the child, leading to tension among caregivers when conflicting values negatively influenced child feeding practices.

The way that my in‐law side eats right, makes me really cringe. (…) They have no (health) consciousness. So, therefore, my kid also thinks that *dabao* (takeaway) food taste nicer (…); it really frustrates me. (P03)

#### 3.2.2 Indulgence With Sugary Treats

Parents were typically stricter than grandparents with regard to restricting the child's intake of sugary snacks. Feeding rules regarding these sugary snacks were often established by parents and grandparents were expected to adhere to them. However, some grandparents felt that parental rules were overly restrictive and that exposure of the young child to a variety of flavours and food was important.

Sometimes I feel like they (parents) are a little too strict, I think. Never mind, let them try, now that they're young. (GP04)

While some grandparents conformed to parental regulations despite differing beliefs, others disregarded their instructions and secretly fed the child ‘prohibited’ sugary treats, much to the frustration of parents.

I mean I've caught her (grandmother) doing it like secretly. Like “oh, don't tell your mother I'm gonna give you this sweet treat”. (P03)

Some parents shared how they showed compromise towards grandparents to maintain peaceful family relations, in contrast to other involved caregivers (such as domestic helpers).

For my parents, if I can close one eye, I just close it. But for my helper I gave her the rules that look, no snacks for them, no soft drinks. (P02)

Grandparents perceived their role to be more indulgent and different from parents, who were depicted as the child's disciplinarians. This gave them the privilege to express love and dote on their grandchild, which included occasionally indulging the child with sugary treats. This feeding practice garnered them favour with the child and reinforced their special identity as grandparents.

You know what grandparents are like, we are not there to discipline and say no all the time. We want the grandchild to always remember their grandparents like great, great people. (GP04)

Infrequent acts of indulgence were not seen to cause disruption to the child's feeding schedule or significant detriment to the child's dental health. Grandparents also reported that they practiced moderation and were judicious with offering sugary treats, taking into consideration the importance of a healthy balanced diet.

I mean, it doesn't do very much harm… A few months one pancake (laughs). (GP04)

On the contrary, in other families, some grandparents had strong personal beliefs in nutritional and dental health and took a more active role in regulating sugary snack intake and educating the child about healthy food choices and feeding habits. In one family, grandparents were reported to be stricter than the parents in controlling sugary snack consumption.

Usually they (grandparents) criticise my snacks. (…) They're very health conscious, so they don't like to feed them with snacks. (P02)

### 3.3 Impact of New Information Sources Versus Grandparental Input

Parents often used information from social media and peers, while grandparents relied on past experiences for child feeding decisions. Secondary caregivers, like domestic helpers and formal childcare providers, had little influence on these decisions. Domestic helpers only carried out caregivers' instructions on food and milk preparation and feeding, often with clear restrictions and regulations imposed to ensure their adherence. Childcare centres were not mentioned as influences on feeding choices by the participants.

Parents independently sought out child nutritional information from online sources, and in some cases, even from scientific articles and journals. They did not always readily accept grandparental advice, which was sometimes considered outdated. In areas where grandmothers did not have a rich personal experience, such as breastfeeding, they were not confident in giving advice and this led to mothers depending on online resources and peer support groups instead.

I like evidence‐based stuff. (…) I will need to do my own research to think about whether that anecdotal thing has any weight to it. (P04)

Grandparents remarked about their limited influence on child feeding practices due to parents' easy access to online sources of information, unlike in the past where first‐time parents mainly relied mostly on grandparents for guidance.

In fact, now I think it's a reversal. Grandparents cannot influence very much. (…) They (Parents) get so much information on the net. They are over flooded with information. (GP04)

Moreover, grandparents often chose not to interfere with parental decision‐making. When disagreements about child feeding practices arose, grandparents sometimes negotiated with parents but ultimately felt that they did not have the authority to overrule parental decisions.

But because ultimately, my grandchild is their child, I feel that I don't have the authority to say, “You must follow what I say”. (GP05)

Although it may appear that parents depended heavily on social media for information, grandmothers still had considerable impact on child feeding practices due to their active involvement in food preparation and feeding. First‐time mothers who felt lacking in practical skills frequently relied on grandmothers for guidance, which was considered more helpful than theoretical information parents acquired from other sources. In some families, parents entrusted child feeding entirely to grandmothers who were perceived to be more experienced. Practical reasons such as busy parental work schedules also resulted in delegation of child feeding responsibilities to grandmothers who had more free time.

‘I mean my mother‐in‐law is very big on feeding (the child) right. So, I just trust her and then she cooks, and yeah, it's all very healthy, I'm sure’. (P06)

## 4 Discussion

Our study found that generational mindsets and beliefs towards milk feeding practices were distinctively different, which led to conflicting input by intergenerational caregivers as to what was best for the child. The desire and practice of breastfeeding was more common among new mothers, while formula milk feeding was the norm in the grandparents' era. These intergenerational differences may be attributed to the changing socio‐economic environment and nutrition messaging in Singapore over time. Grandparents were new parents around the 1980s, a period when Singapore was undergoing rapid industrialisation and economic development. The influx of women into the workforce as well as changing lifestyles were some of the factors contributing to the historically low breastfeeding rates among mothers in Singapore at that time [21]. Moreover, breastfeeding support was not well established and expressed breastfeeding was not common as user‐friendly electric breast pumps had yet to be developed and popularised [22]. The resultant shift towards formula milk feeding in the grandparents' generation, combined with aggressive formula marketing in an era when advertising of breastmilk substitutes was not fully regulated [21], may have strengthened grandparents' beliefs that formula milk is equal (or even superior) to breastmilk and that it was an integral component of early childhood nutrition.

Encouragingly, breastfeeding rates in Singapore have steadily increased since the 1990s, which is a likely result of substantial advocacy for breastfeeding on both global and local fronts [23] and regulatory policies on marketing of breastmilk substitutes established by the World Health Organization in 1981 [24]. In our study, grandparents acknowledged the benefits of breastfeeding but their support for breastfeeding was short‐lived due to concerns about the stress and inconvenience of breastmilk expression on working mothers. More should be done to cultivate favourable grandparental attitudes towards breastfeeding which reportedly has positive impacts on mothers' breastfeeding practices [21, 25]. Promoting breastfeeding in infancy not only has significant benefits on the child's general health but also may protect against ECC in the first year of life [26].

Grandparental influence on milk feeding practices expanded beyond infancy to early childhood. Grandparents in our study were more likely to promote prolonged formula milk feeding and encourage continued comfort feeding practices with the milk bottle past infancy. Grandparents' uncertainty about the appropriateness of cow's milk or perception that it has insufficient nutritional value also stemmed from their strong‐rooted belief that formula milk is essential and superior to other milk alternatives in early childhood. However, formula milk is more cariogenic than cow's milk [27], and inappropriate use or an overreliance on formula milk may contribute to cariogenic feeding patterns such as bedtime bottle‐feeding and frequent between‐meal milk feeds [28].

Some studies support the perception that grandparents promote cariogenic eating practices, such as indulging their grandchildren with discretionary foods and sugary treats [15, 29, 30, 31]. These behaviours reportedly arise from ‘grandparental privilege’ and the perceptions of their caregiving role [15, 19]. While this was a common phenomenon among the families in this study, grandparents still appeared to practice discernment and moderation when offering their grandchildren cariogenic snacks. Moreover, grandparents also engaged in positive feeding practices such as providing nutritious home‐cooked meals and educating their grandchildren about nutrition and food values. This mirrors other studies that depict a more balanced approach that grandparents took towards child feeding [19]. We postulate that the mixed findings in the literature may be partially attributed to the level of caregiver involvement. In this study, grandparents were mostly regular and committed caregivers, who have been reported to be less indulgent and more aligned to parental feeding practices compared to casual grandparent caregivers [12, 19].

Based on this study's findings, it appears that the interdependence between parent and grandparent caregivers has significant influence on child feeding practices. Intergenerational differences in child feeding beliefs and practices emerged as a result of changing feeding trends, differing sources of information and perceptions of their caregiving roles. Conflicting opinions from involved caregivers led to a difficult negotiation process. When a consensus could not be reached, compromises were made in the hope of maintaining ‘peace’ within the family. However, unresolved differences remained as barriers to healthy feeding practices and encouraged the persistence of cariogenic habits. This, in turn, worsened caregivers' frustrations and intergenerational relationships, adding to the stress and burden already associated with child feeding. Burgette et al. explored the complex intergenerational interactions and its effect on cariogenic dietary practices [16]. The authors identified social factors such as frequency of interaction between grandparents and children, the degree of dependency of mothers on grandparents for childcare and strengths of the relationship between mothers and grandparents that influence mothers' decision to address grandparents' cariogenic food choice for the children. Future oral health promotion should consider strategies that strengthen family cohesion as a foundation to the successful adoption of healthy child feeding practices, especially in intergenerational families.

Studies indicate that caregiver stress related to child feeding affects feeding practices and child health outcomes [32, 33]. A complex interplay of factors is present, such as the pressure to follow feeding guidelines, the difficulty of balancing work and family duties, and the lack of accessible support and resources [34]. Moreover, studies reveal the feelings of caregiver guilt and shame over child feeding as mothers struggle to balance between ‘being a good mother’ and realities of daily lives [33, 34]. Likewise, in the dental context, caregiver stress can lead to them choosing more convenient but cariogenic feeding methods, such as bedtime milk feeding to initiate sleep. Knowing that these practices can cause harm to the child's teeth and health, negative caregiver emotions may emerge and worsen stress and coping abilities. To address these issues, it is essential to provide comprehensive support to caregivers which may include accessible professional guidance, peer support groups and creating supportive environments in childcare settings. Moreover, interventions should aim to reduce the stigma and guilt associated with feeding challenges and promote a responsive feeding style that also considers the caregiver's well‐being [32, 33, 34, 35]. Future research should investigate the impact of caregiver stress on cariogenic feeding practices and identify effective strategies to alleviate this stress.

Healthcare professionals can play a crucial role in helping intergenerational caregivers bridge their differences and reach a consensus on healthy feeding practices. Dental health practitioners should actively encourage grandparent caregivers to attend dental visits alongside parents, ensuring that preventive advice is communicated to the entire family. By understanding the context behind certain generational mindsets and beliefs, practitioners can better address misconceptions about child feeding practices. This can be achieved by providing up‐to‐date nutritional information to caregivers, such as clarifying the nutritional content of different types of milk and appropriate transitions in milk feeding. While practitioners should acknowledge grandparents' natural inclination to indulge their grandchildren, they can suggest alternative practices, such as using non‐food treats (toys, prizes, etc.) to reward the child and reinforce a positive intergenerational bond.

On a public health level, policymakers should recognise grandparents as key stakeholders in promoting children's health and ensure their involvement in public health initiatives, especially in societies where intergenerational caregiving is prevalent. Engaging parents and grandparents in co‐designing public health programmes can help address their needs and concerns, leading to person‐centred solutions. In the local context, preventive campaigns promoting oral health and healthy nutritional practices are still primarily targeted at parents and mostly disseminated via social media platforms. Our findings indicate that grandparents rely less on social media for information on child feeding compared to parents. Research into the most effective platforms and methods to engage grandparents in fostering healthy child feeding practices is currently lacking and would be a valuable contribution to future research and public policy development.

Findings of this study should be interpreted with consideration of the methodological strengths and limitations. The strength lies in the inclusion of both parents and grandparents which provided a comprehensive portrayal of the child feeding experience within a intergenerational care context. Participants were interviewed separately with assurance of confidentiality which allowed them to share their views freely.

There were some limitations in our study. As the target number was not reached for parents with non‐university degrees and those not engaged in full‐time employment, findings may not have entirely captured the experiences of families with a lower socio‐economic status. Most of the grandparents in our study were retired or not working, which may have made them more readily available for interviews or more involved in childcare. Due to the low number of working grandparents in our sample, their influence on child feeding practices has yet to be fully explored. Although recruitment was not restricted to female caregivers, the recruited participants were mostly female because mothers and grandmothers were the main caregivers in these families. As a first exploratory study in this topic, only Chinese race was included. Perspectives of fathers and other ethnic groups should be explored in future studies. The role and contribution of formal childcare centres and domestic helpers were not explored in depth as this was not the study's primary aim. Although the findings of this study may not be directly applicable to all populations, they could be relevant to countries with modern societies where intergenerational caregiving is prevalent.

This qualitative study adds newer and deeper insights to intergenerational caregivers' perspectives and experiences with child feeding. Grandparents have significant influence in shaping early child feeding practices, and their role in prevention of ECC should not be overlooked. Family‐centric interventions, involving both parents and grandparents, should be designed as part of a holistic approach to the promotion of healthy child feeding practices in intergenerational families.

## Author Contributions

**Su Lin Quak:** data curation, formal analysis, project administration and writing – original draft. **Huei Jinn Tong:** conceptualization, writing – review and editing and supervision. **Catherine Hsu Ling Hong:** writing – review and editing and supervision. **Mary Foong Fong Chong:** writing – review and editing. **Monty Duggal:** conceptualization and supervision. **Zubair Amin:** formal analysis, project administration, supervision, writing – review and editing. **Xiaoli Gao:** conceptualization, formal analysis, project administration, supervision and writing – review and editing. All authors read and approved the final manuscript.

## Ethics Statement

The study was approved by the National University of Singapore's Institutional Review Board (NUS IRB) (reference no.: 2020/01478).

## Consent

All participants provided written consent to participate in the study. The manuscript does not contain any identifiable information.

## Conflicts of Interest

The authors declare no conflicts of interest.

## Data Availability

The data that support the findings of this study are available on reasonable request from the first author, Su Lin Quak.

## Appendix A COREQ (COnsolidated Criteria for REporting Qualitative Research) Checklist

**Table:**

Topic	Item no.	Guide questions/description	Remarks	Page no.
**Domain 1: Research team and reflexivity**				
*Personal Characteristics*				
Interviewer/facilitator	1	Which author/s conducted the interview or focus group?	The first author (Su Lin Quak) conducted the in‐depth interviews.	6
Credentials	2	What were the researcher's credentials? e.g., PhD, MD	The researchers' credentials are as follows: Su Lin Quak: BDS, MDS Huei Jinn Tong: BDS, MS Catherine Hsu Lin Hong: BDS, MClinDent, MPaedDent Mary Foong Fong Chong: BSc, PhD Monty Duggal: BDS, MDSc, PhD Zubair Amin: MD, MHPE Xiaoli Gao: BDS, MSc, PhD	—
Occupation	3	What was their occupation at the time of the study?	The researchers' occupations are as follows: Su Lin Quak: Postgraduate student Catherine Hsu Ling Hong: Associate Professor Huei Jinn Tong: Adjunct Senior Lecturer Mary Foong Fong ChonAssociate Professor Monty Duggal: Professor Zubair Amin: Associate Professor Xiaoli Gao: Associate Professor	—
Gender	4	Was the researcher male or female?	The researchers' gender are as follows: Female Female Female Female Male Male Female	—
Experience and training	5	What experience or training did the researcher have?	Su Lin Quak is formally trained in qualitative research methods.	6
*Relationship with participants*				
Relationship established	6	Was a relationship established prior to study commencement?	There was no pre‐existing relationship between any of the participants and the interviewer (Su Lin Quak).	6
Participant knowledge of the interviewer	7	What did the participants know about the researcher? e.g., personal goals, reasons for doing the research	None of the participants knew the interviewer Su Lin Quak personally. Su Lin Quak explained the goals of the research during study recruitment.	6
Interviewer characteristics	8	What characteristics were reported about the interviewer/facilitator? e.g., Bias, assumptions, reasons and interests in the research topic	The interviewer introduced herself as a child dentist interested in studying the effect of feeding practices on dental caries.	6
**Domain 2: Study design**				
*Theoretical framework*				
Methodological orientation and Theory	9	What methodological orientation was stated to underpin the study? e.g., grounded theory, discourse analysis etc	A phenomenological research approach was adopted.	1,4
*Participant selection*				
Sampling	10	How were participants selected? e.g., purposive, convenience, consecutive, snowball	Purposive and snowball sampling	4,5
Method of approach	11	How were participants approached? e.g., face‐to‐face, telephone, mail, email	A flyer was disseminated by the referring clinician and interested participants were approached face‐to‐face or over the telephone by a study team member. Participants were also recruited via word‐of‐ mouth from existing study participants as part of the snowball sampling strategy.	5
Sample size	12	How many participants were in the study?	13 families were recruited.	6
Non‐participation	13	How many people refused to participate or dropped out? Reasons?	Of the 25 families screened, 12 were not recruited as they either did not fulfil the inclusion criteria (n=6) or declined participation (n=6).	6
Setting				
Setting of data collection	14	Where was the data collected? e.g., home, clinic, workplace	Data were collected at the families' home (face‐to‐face) or the researcher's workplace via videocall.	6
Presence of non‐participants	15	Was anyone else present besides the participants and researchers?	No one else was present besides the participants and the interviewer (Su Lin Quak).	6
Description of sample	16	What are the important characteristics of the sample? e.g., demographic data, date	There were 13 mothers and 13 grandmothers and one grandfather.	6
Data collection				
Interview guide	17	Were questions, prompts, guides provided by the authors? Was it pilot tested?	There was an interview guide with prompts (Appendix B); this was pilot tested prior to use.	5
Repeat interviews	18	Were repeat interviews carried out? If yes, how many?	No repeat interviews were carried out.	7
Audio/visual recording	19	Did the research use audio or visual recording to collect the data?	Interviews were audio recorded.	6
Field notes	20	Were field notes made during and/or after the interview or focus group?	Field notes were made by Su Lin Quak after every interview. These field notes were taken to capture the interviewer's raw thoughts and reflections, and used to assist in the analysis of the transcribed audio recordings.	6
Duration	21	What was the duration of the interviews or focus group?	Duration of the in‐depth interviews was approximately 60 min.	Appendix A
Data saturation	22	Was data saturation discussed?	Data saturation was discussed in the Methods section.	7
Transcripts returned	23	Were transcripts returned to participants for comment and/or correction?	The transcripts were not returned to participants for comment or correction.	7
**Domain 3: analysis and findings**				
*Data analysis*				
Number of data coders	24	How many data coders coded the data?	One (Su Lin Quak)	6
Description of the coding tree	25	Did authors provide a description of the coding tree?	No	—
Derivation of themes	26	Were themes identified in advance or derived from the data	The themes were derived from the data (inductive thematic analysis).	6
Software	27	What software, if applicable, was used to manage the data?	NVivo version 12 was used to manage the data.	6
Participant checking	28	Did participants provide feedback on the findings?	No, findings were not returned to the participants for feedback.	7
*Reporting*				
Quotations presented	29	Were participant quotations presented to illustrate the themes/findings? Was each quotation identified? e.g., participant number	Yes, quotations were presented and identified by the respondent (parent or grandparent).	7–13
Data and findings consistent	30	Was there consistency between the data presented and the findings?	Yes, there was consistency between the data presented and the findings.	7–13
Clarity of major themes	31	Were major themes clearly presented in the findings?	Yes, major themes were clearly presented in the findings.	7–13
Clarity of minor themes	32	Is there a description of diverse cases or discussion of minor themes?	Yes, there were descriptions of the diverse cases or discussion of minor themes presented.	7–12

## Appendix B Interview Guide

### Introduction

Good morning/afternoon. Thank you for agreeing to the interview. I am *(Name)*, a Master's student in Paediatric Dentistry from the Faculty of Dentistry, National University of Singapore. This interview is part of a research study and it should take about 60 min.

### Objectives of Interview

The objective of the study is to learn more about the influence of intergenerational caregivers on child feeding practices. We will discuss about the child's breastfeeding, bottle feeding, and snacking habits, some of which may affect their dental health. We would like to know more about your experience as one of the few/many caregivers of this child.

A better understanding of the experiences and challenges faced by family caregivers will help in formulating policies to promote healthier feeding practices.

### Confidentiality & Anonymity

The interview will be recorded on an audio device, but all recordings will be kept confidential and will not record your name. Only the research team will have access to the research data. You may withdraw from the interview at any time if you are uncomfortable or for any other reason.

✓ Any questions?

✓ Consent

**A. Section on Caregivers**

*Objective: As an ice‐breaker and to familiarise with the family's caregiving structure*

**Table:**

Topic	Sample Questions
Information about Caregivers	Can you describe: In a typical week, what is the child's schedule like? Who helps to look after him? ○ *Prompt: Living arrangements, childcare centres* ○ *Prompt: Past and present, main and secondary caregivers* 2 How much time do you/other caregivers spend with the child per week? 3 What are their caregiving responsibilities?

**B. Section on Child Feeding Practices and Dental Health**

*Objective: To understand caregiver's perspectives and practices related to child feeding*

**Table:**

Topic	Sample Questions
Milk‐feeding Practices	Can you describe the breastfeeding and bottle feeding practices of this child from birth until now? **Breastfeeding** Can you describe what the latching habits were like? ○ *Prompt: Latching at night, on‐demand habits* What were the reasons for these latching habits? **Bottle‐feeding** Can you describe the child's bottle feeding routine? ○ *Prompt: Duration of use* ○ *Prompt: Content of bottle* ○ *Prompt: Frequency, on‐demand habits* ○ *Prompt: Bottle before sleep or to sleep habits* What are the reasons for these milk habits? What is the purpose of latching/offering the milk bottle?
Snacking habits (Intake of sugary snacks and drinks)	Can you describe what the child's snacking habits are like? ○ *Prompt: Sugary content, frequency, on‐demand* What are the reasons/purpose of offering sugary snacks/drinks? ○ *Prompt: In what situations will the child be offered sugary snacks/drinks? Who offers these sugary snacks/drinks?*
Challenges faced	Were there any milk or snacking habits that you found undesirable? What were the challenges faced with changing undesirable feeding habits? What are the challenges faced with maintaining good dental hygiene or health for the child?
Perspectives of caregivers and Intergenerational differences	What are your views on your child's milk/snacking habits on his/her dental health? ○ *Prompt: How are your views similar or different to the parents/grandparents?* ○ *Prompt: Why do you think there are these similarities/differences?* How are your feeding practices similar or different from the parents/grandparents?

**C. Section on Intergenerational Interactions**

*Objective: To explore the intergenerational interactions, including decision‐making, differences and conflict resolution, and effects on familial relationships*

**Table:**

Topic	Sample Questions
Decision‐making process	Who makes the decisions regarding what kind of milk or snacks the child eats or how the child should be fed? ○ *Prompt: How do you/they come to these decisions? What are the considerations?* Do you receive/give any suggestions regarding what kind of milk or snacks to provide for the child? Are there rules regarding what and how to feed the child? ○ *Prompt: How do the other caregivers respond to these instructions?*
Intergenerational Conflicts and Resolutions	Can you describe any challenges or conflicts you faced with the parents/grandparents regarding the child's milk/snacking practices? How do the caregivers communicate with each other regarding issues with the child's milk/snacking practices? How are you able to come to an agreement or compromise with the parents/grandparents?
Roles	In looking after this child, how would you describe: Your own role/responsibility and The role/responsibility of other caregivers? How much influence/control do you or the parents/grandparents have on the child's milk and snacking habits?
Dynamics	How is it like having many caregivers/being one of many caregivers looking after the child? What are the dynamics/relationship like among the caregivers? Can you share any examples of good/bad experiences related to having other caregivers involved in looking after and feeding the child? What are the advantages and disadvantages about having multigenerational caregivers involved in these child feeding practices? ○ *Follow up: Can you give any real‐life examples?* In the experience of looking after the child together, how has your relationship with the parent/grandparent changed?
